# Gene silencing by RNA interference in *Sarcoptes scabiei*: a molecular tool to identify novel therapeutic targets

**DOI:** 10.1186/s13071-017-2226-1

**Published:** 2017-06-10

**Authors:** Deepani D. Fernando, Edward J. Marr, Martha Zakrzewski, Simone L. Reynolds, Stewart T. G. Burgess, Katja Fischer

**Affiliations:** 1 QIMR Berghofer Medical Research Institute, Infectious Diseases Department, 300 Herston Road, Herston, Brisbane, 4006 Australia; 2 School of Veterinary Sciences, University of Queensland, Gatton, QLD 4343 Australia; 3 Parasitology Division, Moredun Research Institute, Pentlands Science Park, Bush Loan, Edinburgh, Midlothian, Scotland EH26 0PZ UK; 4 Department of Veterinary Pathobiology, Faculty of Veterinary Medicine and Animal Science, University of Peradeniya, Peradeniya, Sri Lanka

**Keywords:** Scabies mites, *Sarcoptes scabiei*, RNA interference, Gene knockdown, Glutathione S-transferase

## Abstract

**Background:**

Scabies is one of the most common and widespread parasitic skin infections globally, affecting a large range of mammals including humans, yet the molecular biology of *Sarcoptes scabiei* is astonishingly understudied. Research has been hampered primarily due to the difficulty of sampling or culturing these obligatory parasitic mites. A further and major impediment to identify and functionally analyse potential therapeutic targets from the recently emerging molecular databases is the lack of appropriate molecular tools.

**Methods:**

We performed standard BLAST based searches of the existing *S. scabiei* genome databases using sequences of genes described to be involved in RNA interference in *Drosophila* and the mite model organism *Tetranychus urticae*. Experimenting with the *S. scabiei* mu-class glutathione S-transferase (*SsGST-mu1*) as a candidate gene we explored the feasibility of gene knockdown in *S. scabiei* by double-stranded RNA-interference (dsRNAi).

**Results:**

We provide here an analysis of the existing *S. scabiei* draft genomes, confirming the presence of a double stranded RNA (dsRNA) - mediated silencing machinery. We report for the first time experimental gene silencing by RNA interference (RNAi) in *S. scabiei.* Non-invasive immersion of *S. scabiei* in dsRNA encoding an *S. scabiei* glutathione S-transferase mu-class 1 enzyme (*SsGST-mu1*) resulted in a 35% reduction in the transcription of the target gene compared to controls.

**Conclusions:**

A series of experiments identified the optimal conditions allowing systemic experimental RNAi without detrimental side effects on mite viability. This technique can now be used to address the key questions on the fundamental aspects of mite biology and pathogenesis, and to assess the potential therapeutic benefits of silencing *S. scabiei* target genes.

**Electronic supplementary material:**

The online version of this article (doi:10.1186/s13071-017-2226-1) contains supplementary material, which is available to authorized users.

## Background

Scabies shows many typical features of a truly neglected tropical disease [1, 2]. It is a skin disease that affects worldwide, primarily economically disadvantaged populations [3–6]. It is common in the tropics and here the initial epidermal skin infections with parasitic mites often escalate into complex secondary infections with mite-associated bacterial pathogens, potentially causing serious subsequent sequelae. Burrowing mites and host scratching responses cause disruption of the skin barrier, thereby providing an entry point for bacteria, which can become invasive, or cause post-infection complications (e.g. post-streptococcal glomerulonephritis and rheumatic heart disease) [7]. The entire scabies mite life-cycle is ‘obligatory parasitic’ [8] as scabies mites are thought to be extremely vulnerable to water loss [9–11]. Environmental reservoirs have not been reported, except for a small proportion of adult mites opportunistically surviving on fomites for a few days [12–14] if under optimal conditions, i.e. high relative humidity and moderate temperatures. Hence treatment of infected hosts is the most direct way to effectively combat the disease. There is no vaccine against scabies, and it is doubtful whether vaccine development is feasible or affordable [15]. Only a few broad spectrum anti-parasitic drugs are currently in use to treat scabies [16, 17], but these often fail to control the disease because of their limited ovicidal activity and their short half-lives [18, 19]. This dictates multiple treatments and combining topical and systemic drugs, which causes considerable management issues [19]. In addition the first evidence of emerging resistance in scabies mites against the most commonly used drugs permethrin [20] and ivermectin [21, 22] have been reported. Importantly, no sensitive and simple molecular diagnostic tools are currently available and this is the main reason for the worldwide deficit in scabies surveillance, as summarised recently [3]. The fact that scabies is not a reportable disease in many countries further impedes the accurate accounting of scabies prevalence. As a consequence the public health burden due to scabies and associated secondary infections is under-recognised worldwide and accordingly, funding for health services to control scabies or for research to gain further knowledge about the disease is extremely limited. As a result, scabies (and mange) remains a small, understudied research field.

Aside from the challenges presented due to a lack in recognition from a funding perspective, scabies is also a difficult disease to study practically. *S. scabiei* resides within the epidermis throughout its entire life-cycle [8] and its water balance appears to be highly adapted to the conditions within the protective matrix of the stratum corneum [10]. Establishing a continuous in vitro culture would require precise mimicking of the epidermal conditions; this has only been accomplished to a limited extend [23]. Currently most molecular and biochemical research of the parasite still relies entirely on sampling of mites from the living host. However, most human as well as animal hosts, if not treated, develop chronic but self-limiting scabies infestations, harbouring relatively few parasites on their bodies (up to 20 in humans [24]). These mites are of microscopic size (< 400 μm in length), and high numbers of parasites are needed for research applications. As a consequence most scabies infections are not suitable for laboratory based parasite studies. For all these reasons mite molecular biology and biochemical research of *S. scabiei* remains a largely unexplored field and has lagged behind within the field of acarology. Consequently, until very recently, few molecular data existed and none of the molecular tools already established in other parasite research fields were available.

To overcome the significant lack of molecular data on scabies a few research groups have attempted to deploy the only substantial parasite material sources available, which are animal and human cases of crusted or Norwegian scabies. Formation of hyperkeratotic skin crusts is a very rare and extreme form of scabies occurring predominantly in immune-compromised individuals (reviewed in [25]). In this condition thousands of mites are contained per gram of skin crust. Crusted scabies is a rare but terrible state of this disease, requiring intensive care and long-term treatment. These rare cases have the potential to provide the only substantial resource of parasites for molecular research, for example samples from two such patients facilitated the generation of the first *S. scabiei* var. *hominis* draft genome database [26]. Furthermore, an in vivo porcine model has been established recently, where localised crust formation can be enforced by the administration of immune suppressing steroids [27]. The development of this model system has allowed for the commencement of a genome [26], transcriptome and proteome project of *S. scabiei* var. *suis* mites [manuscript in progress]. Draft genome and proteome databases of *S. scabiei* var. *canis* have also been established [28, 29]. The generation of these molecular databases will allow us to understand fundamental aspects of mite biology and pathogenesis in order to develop novel control strategies.

In the advent of the establishment of mite multi-omics it is compulsory to develop molecular tools and techniques that allow experimentation, analysis and exploration of the molecular information gained. RNA interference (RNAi) is a reverse genetic tool displaying promising analytical potential to explore gene function [30]. This technology has enabled the analysis of gene function in numerous organisms and could facilitate the systematic selection of potential chemotherapeutic target molecules from the newly established *S. scabiei* molecular data sets. Similar work with other arthropods is underway and RNAi has been previously applied for ticks [31], flies and parasitic copepods (reviewed in [30]). RNAi silencing has been demonstrated in a few mites, including the pest mites *Varroa destructor* (honey bee mite) [32, 33], *Tetranychus urticae* (two-spotted spider mite) [34] and *Dermanysus gallinae* (poultry red mite) [35] and also in *Metaseiulus occidentalis* [36], an agriculturally important biological control agent of plant-feeding pest mites. More recently RNAi has also been tested in Sarcoptiformes mites including the notorious house dust mite *Dermatophagoides pteronyssinus* [37] and the sheep scab mite, *Psoroptes ovis* (E. J. Marr personal communication). Glutathione S-transferase mu1 (*GST-mu1*) has been the gene target of choice for most studies aiming to establish RNAi in mites. GSTs play critical roles in detoxification and have been shown to be involved in the development of acaricide resistance [38–40]. RNAi can be achieved by the introduction of either double-stranded RNA (dsRNA) or small interfering RNA (siRNA) into an organism, provided the organism possesses the principal effectors of the RNAi pathway. RNAi is thought to have evolved as a defence against viruses and is indeed a highly conserved mechanism of genetic regulation present in many eukaryotes (reviewed in [30]). When introduced into a target cell, ds/siRNA is processed and incorporated into the RNA-induced silencing complex (RISC), which leads to the specific cleavage of gene-specific messenger RNA (mRNA) with complementary sequence homology to the ds/siRNA trigger [41]. The phenotype arising from the reduction in gene specific transcription levels caused by RNAi can indicate the function(s) of the protein encoded by the target gene and thereby may suggest its importance to the host organism, as recently summarised for the study of tick physiology [31]. In organisms like *S. scabiei*, for which annotated genomic information does not yet exist, and for which transfection has not been established, RNAi could be a useful technology to inform on the potential of candidate genes as novel intervention targets. To date there has been no report of RNAi being trialled in scabies mites, possibly due to the above mentioned limitations of sampling, their miniscule size and the difficulty in maintaining them in vitro. We have analysed the recently generated draft genome databases of *S. scabiei* varieties from canine, porcine and human hosts for the presence of the RNAi machinery and report here the basic and typical elements of an RNAi pathway in *S. scabiei*. We also developed a non-invasive RNAi methodology specific for *S. scabiei* and demonstrate the successful experimental gene-silencing of *SsGST-mu1* in *S. scabiei* var. *suis* following immersion in complementary dsRNA.

## Methods

### Reference sequences for screening RNAi pathway components in *Sarcoptes scabiei* genomic databases

The reference sequences of proteins involved in small RNA-mediated silencing in *Tetranychus urticae* were obtained from Grbic et al. [42] and in *Drosophila melanogaster* from NCBI protein and Swissprot databases. The reference identification numbers used are listed in Table 1.

**Table 1:** Reference genes used to search *S. scabiei* databases

Gene family (Organism)	Gene ID
Dicer (*T. urticae*)	tetur07g00990.1.1; tetur19g00520.1.1
Argonaute (*T. urticae*)	tetur02g10560.1.1; tetur02g10570.1.1; tetur02g10580.1.1; tetur04g01190.1.1; tetur09g00620.1.1; tetur09g03140.1.1; tetur20g02910.1.1
Piwi*/Aub/Ago3* (*T. urticae*)	tetur06g03300.1.1; tetur06g05570.1.1; tetur06g05580.1.1; tetur06g05600.1.1; tetur17g03380.1.1; tetur28g00340.1.1; tetur28g00450.1.1
Pasha (*T. urticae*)	tetur36g00220.1.1; tetur36g00250.1.1
Drosha (*T. urticae*)	tetur12g00910.1.1
*GW182* (*T. urticae*)	tetur05g07970.1.1; tetur09g00260.1.1
*VIG* (*T. urticae*)	tetur22g01310.1.1
Loquacious(*T. urticae*)	tetur13g00410.1.1; tetur13g00430.1.1
Exportin-5 (*T. urticae*)	tetur02g00500.1.1; tetur02g00520.1.1
*RdRP* (*T. urticae*)	tetur02g08750.1.1; tetur02g08780.1.1; tetur02g08810.1.1; tetur02g08820.1.1
Dicer (*Drosophila*)	XP_017874916.1
*R2D2* (*Drosophila*)	NP_001285720.1
Argonaute (*Drosophila*)	XP_016037320.1; XP_015024007.1
*GW182* (*Drosophila*)	XP_015024007.1; XP_016037320.1
Piwi (*Drosophila*)	XP_002051133.2; XP_015033122.1; XP_002003896.1
Exportin (*Drosophila*)	XP_017154884.1; XP_002058203.1
dsRNA binding domain, (*Drosophila*)	XP_002016656.1; XP_015022070.1
RNase III domain (*Drosophila*)	XP_002016656.1
*C3PO* (*Tribolum castaneum*)	ABX72055

### Interrogation of *Sarcoptes scabiei* genome databases

*Sarcoptes scabiei* draft reference genome databases for *S. scabiei* var. *canis* [28], *S. scabiei* var. *suis* [26] and *S. scabiei* var. *hominis* [26] were analysed using BLAST software [43] through the National Center for Biotechnology Information (NCBI). To identify molecules involved in the RNAi pathway in *S. scabiei* a standard protein-protein BLAST (BLASTp) search against the predicted protein sequences of the *S. scabiei* var. *canis* draft genome was performed. To screen the *S. scabiei* var. *hominis* and *S. scabiei* var. *suis* databases the BLAST-like alignment tool ‘BLAT’ was used, aligning protein sequences vs translated nucleotide database [44]. Subsequently, genes were predicted in the human and pig mite genomes using the software ‘MAKER’ v2.31.9 [45, 46].

### *Sarcoptes scabiei* mites

*Sarcoptes scabiei* var. *suis* mites were harvested from the previously established porcine animal model [27]. Skin crusts were removed from the host and adult female mites were isolated within 3 h of harvesting.

### Double stranded RNA (dsRNA) preparation

The partial sequence of the target gene *S. scabiei* glutathione S-transferase mu class 1 (*SsGST-mu1*) (GenBank GQ214688.1) consisting of 335 bp and the negative control gene *LacZ* of *Escherichia coli* strain K-12 sub-strain MG1655 (GenBank NC_000913.3) consisting of 319 bp were cloned into the dsRNA production vector pL4440 using *Sac1* and *Sma1* restriction sites for directional cloning. The program ‘Primer3’ [47] was used to design the oligonucleotides for amplification of gene-specific double stranded RNA (dsRNA): *SsGST-mu1* forward primer 5′-TAT GAG CTC ATA TGC TGG CGT GGA TTT CG-3′, *SsGST-mu1* reverse primer 5′-TAT CCC GGG CAT CAG GAA GCT TAG CAA CCA-3′, *LacZ* forward primer 5′-TAT GAG CTC CGT TAC CCA ACT TAA TCG CC-3′, *LacZ* reverse primer 5′-TAT CCC GGG TGT GAG CGA GTA ACA ACC C-3′. Plasmids were linearised with the restriction enzyme *Sac1* (NEB, Ipswich, USA) and subsequently treated with Klenow enzyme (NEB) to remove the overhangs. dsRNA was synthesised using T7 RiboMAX™ Express RNAi system (Promega, Madison, USA) according to the manufacturer’s instructions. dsRNA was quantified by ND-1000 Nano-drop spectrophotometer (Thermo Fischer Scientific, Waltham, USA) and the quality was assessed by agarose gel electrophoresis. The dsRNA was diluted in normal saline (Sigma-Aldrich, St. Louis, USA) to a final concentration of 2.5 μg/μl.

### Fluorescence labelling of dsRNA and assessment of uptake by *S. scabiei*

*SsGST-mu1* dsRNA was labelled using the Silencer™ siRNA labelling kit (Thermo Fischer Scientific). Ten μl labelling dye (Cy3®), 12.5 μg *SsGST-mu1* dsRNA in 15 μl normal saline, 5 μl 10× labelling buffer and 20 μl nuclease free water (Invitrogen, Carlsbad, USA) were combined and incubated at 37 °C for 3 h. To precipitate the dsRNA 5 μl 5 M NaCl and 125 μl cold 100% ethanol were mixed with the reaction and incubated 30 min at -20 °C. The dsRNA was pelleted by centrifugation at 18,400×*g* for 20 min at 4 °C, washed with 70% ethanol and centrifuged for 5 min at the room temperature, air - dried and resuspended in 10 μl of nuclease free normal saline (Sigma-Aldrich). Successful labelling was indicated by pink staining of the pellet and confirmed by agarose gel electrophoresis.

### dsRNA administration to *S. scabiei*

Ten adult female mites were completely immersed in 10 μl (1.25 μg/μl) of fluorescently labelled dsRNA or unlabelled dsRNA on a detached cap of snaplock microcentrifuge tube (Axygen, Corning, USA) and incubated at 4 °C for 24 h. Mites were washed 3 times with molecular grade water (Invitrogen) and immobilised for imaging on a benzyl benzoate coated Petri dish. Z stack images (30–60 slices) of mites that had ingested Cy3® labelled dsRNA were captured using a Zeiss 780-NLO confocal microscope with a 20× Plan-Apochromat (NA 0.8) lens. Cy3® was excited using a 561 nm laser attenuated 97% and emitted light (562 nm to 642 nm) was captured using a GaAsP array detector. Images are presented as a single representative slice from each mite processed using Zen software (Carl Zeiss AG, Oberkochen, Germany).

### RNA extraction and quantification

A series of 8 experiments was conducted to establish the optimal conditions for RNA interference (RNAi) in *S. scabiei* var. *suis* (Table 2). Every experiment involved 5 biological replicates. For each replicate 60 adult female mites were isolated from a scabetic skin crust within 3 h of removal from the host, randomly divided into 2 groups of 30 mites and completely immersed either in 20 μl (2.5 μg/μl) of *SsGST-mu1* (test group) or *LacZ* dsRNA (control group). In total 2400 individual mites (30 mites per replicate, 5 replicates per treatment group, 2 treatment groups, 8 different conditions) were tested. The incubation conditions and durations of each experiment are further summarised in Table 2.

**Table 2:** Exposure of *S. scabiei* mites to dsRNA under different experimental conditions

Experiment	Incubation				Survival rate (%)
	dsRNA	75% serum	Normal saline	Out of liquid^a^	
1	24 h, 4 °C	na	na	na	100
2	48 h, 4 °C	na	na	na	100
3	72 h, 4 °C	na	na	na	100
4	24 h, 4 °C	24 h, RT	na	na	93
5	24 h, 4 °C	48 h, RT^b^	na	na	100
6	24 h, 4 °C	na	24 h, RT	na	100
7	24 h, 4 °C	24 h, RT	24 h, RT	na	100
8	24 h, 4 °C	na	na	48 h, RT^a^	26

*Abbreviations*: na, not applicable; RT, room temperature

^a^dsRNA was removed and mites were kept in humidifier chamber

^b^Heat-inactivated serum was replaced after first 24 h

Mites were examined periodically for signs of viability and dead mites and eggs that had been laid during incubation were removed from the solution. Following incubation, living mites were washed 3 times with molecular grade water (Invitrogen) and homogenised in a ZR BashingBead™ lysis tube containing 800 μl RNA Lysis buffer (Zymo Research, Irvine, USA) using a Precellys® homogeniser (Bertin, Montigny le Bretonneux, France) with 3 cycles of 3400× *g* for 23 s including 60 s interval in between cycles at 4 °C. Lysates were centrifuged at 12,000×*g* for 1 min and the supernatant was transferred to a Zymo-Spin™ IIIC column (Zymo Research). Subsequently, RNA was extracted using the ZR Tissue and Insect RNA MicroPrep™ kit (Zymo Research), including an on-column DNase I treatment step (following the manufacturer’s instructions). RNA was quantified using a Qubit® RNA HS Assay kit (Thermo Fischer Scientific) with the Qubit® Fluorometer (Thermo Fischer Scientific). Quality of the extracted RNA was assessed using a Qsep100 system with Q-Analyzer™ software (BiOptic, La Canada Flintridge, USA).

### Quantitative reverse transcription PCR (RT-qPCR)

For the qPCR assessment of gene silencing at the transcriptional level following immersion of mites in dsRNA gene specific primers were designed using Primer3 [47]. To amplify a gene specific fragment of 198 bp of the *S. scabiei* glutathione S-transferase mu-class 1 gene the *SsGST-mu1* qPCR forward primer 5′-TGG CCC GAA TCT GTT ACG AT-3′ and the *SsGST-mu1* qPCR reverse primer 5′-TGG TGA AAA TTT CTG GTG CAA A-3′ were used. To amplify a 181 bp fragment of the *S. scabiei* Elongation Factor 1 alpha gene the *SsEF1α* qPCR forward primer 5′-TTG GCT TAT ACC TTG GGT GTG-3′ and the *SsEF1α* qPCR reverse primer 5′-CAC CGT TCC ATC CAG AGA TT-3′ were used [48].

cDNA was synthesised from 250 ng of total RNA using Superscript® II reverse transcriptase (Thermo Fischer Scientific) and Oligo(dT)_20_ primer (Thermo Fischer Scientific). Gene transcription was quantified using the CFX384 Touch™ Real-Time PCR detection system (Bio-Rad, Hercules, USA) in triplicate reactions per biological replicate of each treatment group. qPCR was performed in 10 μl reactions, consisting of 5 μl LightCycler® 480 SYBR Green I Mater (Roche, Dee Why, Australia), 1 μl cDNA, 0.1 μl each primer (50 μM) and 3.8 μl molecular grade water (Invitrogen). qPCR cycling times were 95 °C for 10 min followed by 45 cycles of 95 °C for 10 s, 50 °C for 10 s and 72 °C for 10 s. Melt curve analysis was performed with the following cycle conditions: 95 °C for 5 s, 65 °C for 5 s and 95 °C for 5 s. qPCR standard curves with 90–110% reaction efficiency were consistently achieved using 10^9^ copies/μl plasmid template DNA within the range of 10^2^–10^9^ copies/μl. Average target gene transcription was normalised to the average transcription of the constitutively expressed reference housekeeping gene *S. scabiei* Elongation Factor 1 alpha (*SsEF1 α*) [48].

Statistical differences in the mean transcription of the target gene between treatment group and control group was calculated with Student’s *t*-test (*P*-value of <0.05 considered significant), using GraphPad Prism 7 (GraphPad Software Inc., USA).

## Results

### Identification of genes involved in eukaryotic small RNA-mediated silencing in *Sarcoptes scabiei* mite genomic databases

BLAST analyses were performed on the *S. scabiei* var. *canis* genome database [28], on three *S. scabiei* var. *suis* genome databases [26] and two *S. scabiei* var. *hominis* genome databases [26]. Each database contained over 10,000 annotated gene features and the CEGMA estimated completeness ranged from 93 to 99%. For each BLAST result the best hit was selected. Domain predictions were obtained using NCBI domain prediction tools. The results are listed in Table 3 and respective gene and scaffold IDs are provided in Additional file 1: Table S1. No matches were obtained for Piwi, *C3PO* and the Dicer-2 cofactor *R2D2*.

**Table 3:** *S. scabiei* genes involved in RNA interference

RNAi pathway gene	Protein function	Databases		
		*S. scabiei* var. *hominis*	*S. scabiei* var. *suis*	*S. scabiei* var. *canis*
Exportin	Nuclear export of shRNA and pre-miRNAs. [72]	+	+	+
Drosha	A nuclear RNase III that cleaves primary miRNAs (pri-miRNAs) to release hairpin-shaped pre-miRNAs. These are subsequently cut by the cytoplasmic RNase III Dicer to generate mature miRNAs. [73–75]	+	+	+
Dicer	Cleaves dsRNA and pre-miRNA into siRNA and miRNA, respectively. Facilitates the activation of RISC. [50]	+	+	+
Pasha	Essential cofactor for Drosha playing a central role in binding single-stranded fragments of the pri-mRNA. [74]	+	+	+^a^
Loquacious	Cofactor of dicer that is important for enabling the incorporation of dsRNA in silencing complexes. It mediates miRNA biogenesis and, thereby, the expression of genes regulated by miRNAs. [76]	+	+	+
Argonaute	Argonaute proteins bind to different classes of small non-cording RNAs (miRNA, siRNA and piRNAs) which leads to mRNA cleavage and subsequent silencing. [77]	+	+	+
*RdRP*	RNA-dependent RNA polymerase that amplifies miRNAs and small temporal RNAs to produce dsRNA which are then cleaved by the enzyme Dicer to produce siRNAs targeting mRNAs for silencing. [78]	+	+	−
*VIG*	Argonaute related protein in RISC that has role in RNA binding and nuclease activity. [79]	+	+	−
*GW182*	Promotes target silencing by repressing translation and enhancing mRNA turnover, precise mechanism of action unknown. [80]	−	+^a^	−
Piwi	Piwi proteins or piwi domains hydrolyse ssRNA. [81]	−	−	−
*C3PO*	Endoribonuclease that activates the RISC. [82]	−	−	−
*R2D2*	Co factor of dicer2 directing strand specific incorporation of the siRNA. [53, 83]	−	−	−

*Key*: +, denotes genes that were identified in the respective *S. scabiei* database; −, denotes genes that were not detected in the respective *S. scabiei* database

^a^Incomplete gene sequence was identified

### Uptake of fluorescently labelled dsRNA by *S. scabiei* Var. *suis*

**Fig. 1:**
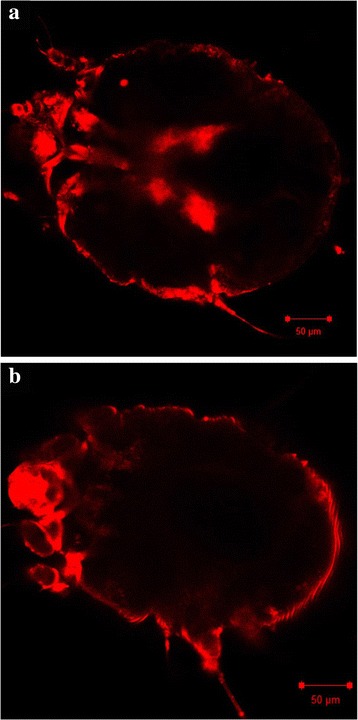
Fluorescence-labelled dsRNA uptake in *Sarcoptes scabiei* var. *suis.* Mites were immersed for 24 h at 4 °C in (**a**) Cy®3-labelled *SsGST*-mu1 dsRNA or (**b**) Unlabelled *SsGST-mu1* dsRNA, and subsequently visualised by confocal microscopy at 20× magnification and excitation wavelength 561 nm. *Scale-bars*: 50 μm

We successfully established a suitable method to introduce dsRNA into *S. scabiei*, adapted from a previously published protocol to silence genes in house dust mites [37]. After 24 h incubation of mites at 4 °C with fluorescently labelled *SsGST-mu1* dsRNA at 1.25 μg/μl, fluorescence was clearly visible in the mite gut (Fig. 1a), indicating oral uptake. Control group mites incubated with either unlabelled *SsGST-mu1* dsRNA (Fig. 1b) did not exhibit fluorescence in their gut. This demonstrated that dsRNA can readily be delivered into the *S. scabiei* through non-invasive immersion. Mites in both groups exhibited auto-fluorescence across their body surface, due to the presence of the auto-fluorescent compounds pteridines and resilin in their cuticle; a feature commonly observed in arthropods when the cuticle is exposed to UV light [49].

### Efficiency and duration of gene knockdown in *S. scabiei*

**Fig. 2:**
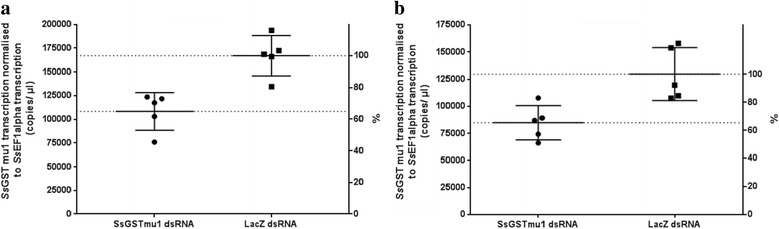
Silencing of the *SsGST-mu1* gene in the scabies mite *S. scabiei* by RNAi. The transcription of the *SsGST-mu1* gene was reduced by 35.14% (*t* = 4.509, *P* = 0.0020) and 34.63% (*t* = 3.45, *P* = 0.0110) in mites treated with *SsGST-mu1* dsRNA. 24 h dsRNA incubation was followed by 24 h (**a**) or 48 h (**b**) serum incubation. The mean *SsGST-mu1* gene transcription normalised to *SsEF1α* gene transcription was determined by qPCR. Depicted is the calculated copy number per μl of cDNA (generated from 250 ng of total RNA template) in preparations from mites that were immersed in *SsGST-mu1* dsRNA (treatment) or *LacZ* dsRNA (control). *N* = 5 × 30 mites for each group (treatment or control). Error bars indicate mean ± SEM

*SsGST-mu1* gene transcription in mites that had been treated with *SsGST-mu1* dsRNA (test group) was significantly (*P* < 0.05) reduced by about 35%, in comparison to the *LacZ* dsRNA-treated control group (Fig. 2), when mites were incubated in dsRNA at 4 °C for 24 h, followed by incubation with 75% heat inactivated serum for another 24 h (*t* = 4.509, *P* = 0.0020) (experiment 4, Fig. 2a) or 48 h (*t* = 3.45, *P* = 0.0110) (experiment 5, Fig. 2b). Repeats of these experiments confirmed the reproducibility of the above key finding. Direct and continuous serum incubation post exposure to RNA seemed essential for gene silencing to occur, as no significant reduction in the *SsGST-mu1* gene transcription was observed if the mites were solely incubated in dsRNA (experiments 1–3), when dsRNA incubation was followed by 24 h normal saline incubation (experiment 6) or when dsRNA incubation was followed by 24 h serum incubation and subsequent 24 h of normal saline incubation (experiment 7). Mite survival data under the different experimental conditions are provided in Table 2. Generally, mites were found alive after immersion in liquid over the maximum tested period of 72 h, but irrespective of provided humidity, mites died if not kept in liquid (experiment 8). Notably, under the conditions of experiments 1–7 egg production seemed to be unaffected, as freshly laid mite eggs were observed.

## Discussion

### Establishing RNAi in *S. scabiei*

RNAi has become a widely used tool to knock down and analyse the function of genes, especially in organisms where in vitro culture systems are absent and the systematic generation of mutants is not feasible. RNAi is therefore a highly attractive experimental approach to test potential drug and vaccine targets in the scabies mite in vitro and in a relatively short time frame, as dictated by the short ex vivo live span of the mite.

Eukaryotic small RNA-mediated silencing involves three essential sets of molecules: small RNAs, Dicer enzymes and Argonaute proteins. Within the nucleus, MicroRNA genes are transcribed into primary microRNAs (pri-miRNAs) and processed by Drosha to release hairpin-shaped pre-miRNAs which are exported by an exportin to the cytoplasm. The ribonuclease III enzyme Dicer excises the small RNAs from their precursors, thereby initiating the RNAi pathway by generating the active short interfering RNA (siRNA). dsRNA binding domain proteins (dsRBD) Pasha, Loquacious, and R2D2 are cofactors for processing events. Silencing is then effected by the RNA-induced silencing complex (RISC) and its RNaseH core enzyme Argonaute. The siRNA is unwound during RISC assembly. When a matching mRNA assembles with the siRNA nucleolytic degradation of the targeted mRNA by the RNaseH enzyme Argonaute occurs causing translational gene silencing [41, 50].

We have assembled here substantial genomic evidence of a complete RNAi machinery present in the *S. scabiei*. Notably, the genes for Piwi, *C3PO* and the *R2D2* were not detected, which does not necessarily imply that the gene is absent in the *S. scabiei*, as the *S. scabiei* databases are not complete and annotations are still underway. Piwi for example has been found in the genomes of *P. ovis* [30] and *T. urticae* [42] and may indeed be found once *S. scabiei* genome analysis has further progressed.

Our demonstration of experimental gene silencing by means of RNAi manifests the presence of a classical functional gene silencing pathway triggered by dsRNA. Experimental delivery of dsRNA can present a challenge. Injecting dsRNA directly into eggs, embryos or adult stages has been efficient in insects and ticks (reviewed in [30]) but is not easily achieved in small mites. Virus-mediated methods have been trialled as another way to overexpress dsRNA [51]. In some but not all mites dsRNA can be introduced simply and efficiently by feeding [52] which allows the dsRNA to enter cells and induce a systemic RNAi effect. Non-invasive immersion has been demonstrated to be the simplest and most gentle experimental method in the honey bee mite, *V. destructor* [33] and the house dust mite, *D. pteronyssinus* [37]. These mites have an experimental advantage as they are either truly ectoparasitic or free-living, respectively, whereas *S. scabiei* are obligatory living within host tissue and cannot be maintained in culture. Nevertheless in our experiments the mites survived and laid eggs while up to 78 h off the host. This indicates a significant achievement of physiological conditions that will in future studies facilitate the experimental assessment of drug targets in *S. scabiei*. Ingestion of labelled dsRNA, as demonstrated here, is a good indicator that we can successfully target genes in the *S. scabiei* gut.

GST enzymes in insects function in detoxification of insecticides [53]. Increased activity of delta and epsilon class GSTs is linked to resistance to organophosphates, DDT and pyrethroids (reviewed in [54]). GSTs have also been associated with macrocyclic lactone resistance in *T. urticae* [40, 55] and *S. scabiei* [39]. We chose *SsGST-mu1* as the first target to trial gene silencing in *S. scabiei* because this is one of the few *S. scabiei* genes that have been studied at the transcriptional level [39] and GSTs have been the target of choice in previously successful trials of RNAi in other mite species [33, 37]. *GST*s are housekeeping genes and have been found to be equally and constitutively expressed in synganglion, malpighian tubules and in the gut of the honey bee mite [33]. *Sarcoptes scabiei* possess at least six glutathione S-transferase genes (group 8 allergen candidates) [28, 56]. Three of these cluster with mu class *GST*s, and the remaining are more related to the delta/epsilon classes of insects, which are of particular interest in the assessment of drug resistance [56]. By carefully selecting targeted regions within non-conserved regions of the gene, it was possible to differentiate between the *S. scabiei GST*s, thereby preventing off-target effects. We chose the gut-localised *S. scabiei GST-mu1* [57, 58], because the gut is easily accessible and also the most dominant compartment of the mite body. RNAi of *GST* genes should occur in the gut even in the absence of a systemic RNAi machinery. However, *SsGST* has been recently shown to increase in expression after removal of the parasite from the host (Kate Mounsey, personal communication). In this respect the 35% reduction of *GST* transmission we measured in experiments 4 and 5 indicates a strong RNAi effect. The silencing of other gene targets under these conditions may be even more profound.

The incubation with serum post dsRNA exposure seems to be crucial. Serum was heat-inactivated to remove nucleases and also to denature the antibodies and complement factors. The addition of serum may have provided a more conducive environment for the mites, mimicking conditions within the host epidermis (pH, ionic strength) ideal to perform physiological functions. In addition, the serum may also have provided a food source for the mites. In the predatory mite *M. occidentalis*, RNAi could only be demonstrated after feeding mites on their hosts *T. urticae* [36]. Similarly it could be that *S. scabiei* also need food in order to successfully initiate the RNAi mechanism.

### Future directions

This demonstration of RNAi in *S. scabiei* is well-timed with the recent publications of the complete mitochondrial genome [59] and nuclear draft genomes [26, 28] of this parasite. In addition transcriptome and microbiome data are underway, altogether providing an abundance of new information that might enable high throughput genome-wide RNAi studies. An interesting focus point is parasite-encoded proteases. Many of these are essential for regulating interactions between parasites and their hosts and, thus, are attractive anti-parasitic drug and/or vaccine targets. Burrowing scabies mites feed in the epidermis [60] and ingest a multitude of diverse host proteins. The feeding success of scabies mites depends on their ability to digest epidermal and plasma components and locally suppress the host complement and coagulation systems by releasing pharmacologically active proteins [61]. We have accumulated extensive data showing that *S. scabiei* express excretory gut proteins involved in these roles [25, 62], including families of active mite cysteine proteases [63], an active serine protease [64], proteolytically inactive serine protease paralogs with novel host complement-inhibitory functions [65–67] and protease inhibitors [68]. Following the optimisation on *SsGST-mu1* and L*acZ*-dsRNA as control, we aim to apply RNAi to silence single genes of potential drug targets, i.e. *SsSar s 3* [64] and *SsAP* [69]. Validation of the gene knockdown in mites could be performed by the established quantitative real-time PCR methods and at the protein level by previously established enzymatic assays and by immunohistology, using established antisera [64, 70]. Using the established porcine scabies model [27] it may also be possible to monitor phenotypic effects and mite survival in situ in the crusted pig skin lesion. As double stranded RNA proved here to be stable over days it might be worthwhile to trial topical treatment [71] with double stranded RNA targeting a gene essential for mite survival, with the aim to reduce mite numbers in an acute infection.

## Conclusion

This demonstration of a significant reduction in the transcription of the *Ss GST-mu1* gene represents the first published account of RNAi in *S. scabiei* and breaks the ground for future RNAi studies in this parasite. Scabies is of considerable medical relevance in particular with regards to complications due to mite associated secondary infections. With current chemotherapeutic control being not enough, the present RNAi approach has the potential to facilitate future research towards developing novel intervention methods.

## Additional file

Additional file 1: Table S1. Gene IDs and scaffold IDs of *S. scabiei* genes involved in RNAi. (DOCX 20 kb)

## Abbreviations

dsRNA: Double stranded ribonucleic acid
qPCR: Quantitative polymerase chain reaction
RNAi: Ribonucleic acid interference
*SsGST-mu1*: *Sarcoptes scabiei* glutathione S-transferase mu-class 1
*SsEF1 α*: *Sarcoptes scabiei* elongation factor 1 alpha
