# Supplementary material for: Gene silencing by RNA interference in Sarcoptes scabiei: a molecular tool to identify novel therapeutic targets
Source: Parasit Vectors. 2017 Jun 10;10:289. doi: 10.1186/s13071-017-2226-1 (PMC5466799; doi:10.1186/s13071-017-2226-1)
Supplement: Additional file 1: Table S1. — Gene IDs and scaffold IDs of S. scabiei genes involved in RNAi. (DOCX 20 kb) [file 13071_2017_2226_MOESM1_ESM.docx]

**Table S1: Gene IDs and scaffold IDs of *S. scabiei* genes involved in RNAi**

|  | dog | Pig unwashed | Pig washed2 | Pig washed3 | Patient A | Patient B |
| --- | --- | --- | --- | --- | --- | --- |
| argonaute | KPM09009.1 | snap-scaffold36952_cov147-processed-gene-0.2-mRNA-1 | snap-scaffold4000_cov96-processed-gene-0.9-mRNA-1 | snap-scaffold485_cov164-processed-gene-0.63-mRNA-1 | snap-scaffold2535_cov62-processed-gene-1.47-mRNA-1 | snap-scaffold1263_cov114-processed-gene-1.7-mRNA-1 |
| dicer | KPM03314.1 | maker-scaffold5043_cov152-augustus-gene-0.6-mRNA-1 | maker-scaffold14598_cov102-augustus-gene-0.20-mRNA-1 | maker-scaffold496_cov169-augustus-gene-0.32-mRNA-1 | maker-scaffold18571_cov61-augustus-gene-0.10-mRNA-1 | maker-scaffold20923_cov124-augustus-gene-0.26-mRNA-1 |
| drosha | KPM02152.1 | augustus-scaffold36969_cov159-processed-gene-0.4-mRNA-1 | augustus-scaffold1678_cov97-processed-gene-0.36-mRNA-1 | augustus-scaffold91_cov161-processed-gene-0.66-mRNA-1 | augustus-scaffold52_cov65-processed-gene-2.21-mRNA-1 | augustus-scaffold2594_cov114-processed-gene-0.117-mRNA-1 |
| exportin | KPM07117.1 | maker-scaffold42841_cov162-augustus-gene-0.6-mRNA-1, maker-scaffold8066_cov152-snap-gene-0.4-mRNA-1 | maker-scaffold3207_cov91-augustus-gene-0.8-mRNA-1 | maker-scaffold1736_cov152-snap-gene-0.24-mRNA-1 | maker-scaffold7584_cov61-augustus-gene-0.20-mRNA-1 | maker-scaffold16643_cov108-snap-gene-0.30-mRNA-1 |
| gw182 | KPM09552.1, KPM09553.1 | maker-scaffold36641_cov153-augustus-gene-0.14-mRNA-1, maker-scaffold36641_cov153-augustus-gene-0.13-mRNA-1 | maker-scaffold20295_cov101-augustus-gene-0.59-mRNA-1, maker-scaffold20295_cov101-augustus-gene-0.60-mRNA-1 | maker-scaffold4029_cov168-augustus-gene-0.41-mRNA-1, maker-scaffold4029_cov168-augustus-gene-0.42-mRNA-1 | maker-scaffold9510_cov61-augustus-gene-0.3-mRNA-1 | maker-scaffold641_cov111-augustus-gene-0.61-mRNA-1, maker-scaffold641_cov111-augustus-gene-0.62-mRNA-1 |
| loquacious | KPM11602.1 | maker-scaffold3744_cov149-exonerate_protein2genome-gene-0.7-mRNA-1 | augustus-scaffold1471_cov98-processed-gene-0.121-mRNA-1 | augustus-scaffold3276_cov168-processed-gene-0.0-mRNA-1 | augustus-scaffold5496_cov64-processed-gene-0.0-mRNA-1 | augustus-scaffold8834_cov124-processed-gene-0.68-mRNA-1 |
| pasha | KPM06480.1 | snap-scaffold36539_cov157-processed-gene-0.12-mRNA-1 | snap-scaffold3079_cov98-processed-gene-2.70-mRNA-1 | snap-scaffold779_cov168-processed-gene-0.26-mRNA-1 | snap-scaffold359_cov63-processed-gene-0.35-mRNA-1 | snap-scaffold1511_cov118-processed-gene-0.74-mRNA-1 |
| rdrp | KPM11269.1 | maker-scaffold16727_cov156-snap-gene-0.1-mRNA-1 | maker-scaffold12035_cov96-snap-gene-0.6-mRNA-1 | maker-scaffold15276_cov167-snap-gene-0.4-mRNA-1 | maker-scaffold7952_cov63-snap-gene-0.10-mRNA-1 | maker-scaffold6466_cov102-snap-gene-0.16-mRNA-1 |
| vig | KPM02238.1 | augustus-scaffold1355_cov153-processed-gene-0.1-mRNA-1 | augustus-scaffold1075_cov93-processed-gene-0.17-mRNA-1 | augustus-scaffold25442_cov164-processed-gene-0.10-mRNA-1 | augustus-scaffold8276_cov62-processed-gene-0.115-mRNA-1 | augustus-scaffold108_cov112-processed-gene-0.11-mRNA-1 |
